# Mitophagy, Mitochondrial Homeostasis, and Cell Fate

**DOI:** 10.3389/fcell.2020.00467

**Published:** 2020-06-24

**Authors:** Kaili Ma, Guo Chen, Wenhui Li, Oliver Kepp, Yushan Zhu, Quan Chen

**Affiliations:** ^1^State Key Laboratory of Medicinal Chemical Biology, College of Life Sciences, Nankai University, Tianjin, China; ^2^Suzhou Institute of Systems Medicine, Chinese Academy of Medical Sciences & Peking Union Medical College, Beijing, China; ^3^Gustave Roussy Cancer Campus, Villejuif, France; ^4^INSERM, UMR 1138, Centre de Recherche des Cordeliers, Sorbonne Université, Université de Paris, Paris, France

**Keywords:** mitophagy, mitochondrial dynamics, mitochondrial apoptosis, cell fate, mitophagy receptors

## Abstract

Mitochondria are highly plastic and dynamic organelles that have graded responses to the changing cellular, environmental, and developmental cues. Mitochondria undergo constant mitochondrial fission and fusion, mitochondrial biogenesis, and mitophagy, which coordinately control mitochondrial morphology, quantity, quality, turnover, and inheritance. Mitophagy is a cellular process that selectively removes the aged and damaged mitochondria via the specific sequestration and engulfment of mitochondria for subsequent lysosomal degradation. It plays a pivotal role in reinstating cellular homeostasis in normal physiology and conditions of stress. Damaged mitochondria may either instigate innate immunity through the overproduction of ROS or the release of mtDNA, or trigger cell death through the release of cytochrome c and other apoptogenic factors when mitochondria damage is beyond repair. Distinct molecular machineries and signaling pathways are found to regulate these mitochondrial dynamics and behaviors. It is less clear how mitochondrial behaviors are coordinated at molecular levels. BCL2 family proteins interact within family members to regulate mitochondrial outer membrane permeabilization and apoptosis. They were also described as global regulators of mitochondrial homeostasis and mitochondrial fate through their interaction with distinct partners including Drp1, mitofusins, PGAM5, and even LC3 that involved mitochondrial dynamics and behaviors. In this review, we summarize recent findings on molecular pathways governing mitophagy and its coordination with other mitochondrial behaviors, which together determine cellular fate.

## Introduction

Mitochondria are organelles that govern energy transformation and ATP production through the tricarboxylic acid cycle (TCA) and oxidative phosphorylation (OXPHOS). Moreover, mitochondria control redox homeostasis, Ca^2+^ signaling, iron metabolism, innate immunity, and apoptotic cell death (Zorov et al., 2014; Zong et al., 2016; Paul et al., 2017; Pathak and Trebak, 2018). Mitochondria are both the major source and the main targets of reactive oxygen species (ROS). Under homeostatic conditions, mitochondrial ROS serve as retrograde signaling molecules for cell growth (Diebold and Chandel, 2016). However, in conditions of stress or aging, mitochondrial ROS elicit oxidative damage to mitochondrial proteins, lipids, and DNA (mtDNA), causing the malfunction of mitochondria. Dysfunctional mitochondria may produce even more ROS via vicious cycle that further amplify the release of ROS and mtDNA into the cytosol, which in turn can act as instigators of inflammation (Nakahira et al., 2011; Zhou et al., 2011). Non-reparable and severe damage of mitochondria leads to the release from the intermembrane space into the cytosol of cytochrome c and other pro-death factors (Sinha et al., 2013) altogether triggering apoptosis, a specific form of programmed cell death. This process is governed by the BCL2 protein family that integrates apoptotic signals and controls mitochondrial outer membrane permeabilization (MOMP).

Mitochondria are highly dynamic organelles that undergo continuous fission and fusion, constant turnover through mitochondrial biogenesis and mitophagy to maintain mitochondrial morphology, homeostasis, and inheritance. When facing bioenergetic or oxidative challenges, mitochondria exhibit a graded response that involves changes in their morphology and dynamics through the activation of distinct molecular machineries that regulate mitochondrial fission, fusion, mitophagy, and mitochondrial biogenesis. Mitochondrial fission and fusion are tightly regulated by a complex protein machinery involving dynamin 1 like (DNM1L better known as Drp1), mitosfusin 1 (MFN1), mitosfusin 2 (MFN2), and Optic atrophy protein 1 (OPA1) in mammalian cells. Mitochondrial fission was found to contribute to mitochondrial apoptosis and was also suggested to be a prerequisite for mitophagy, while mitochondrial fusion is linked to an increase in mitochondrial metabolism. How these molecular machineries sense cellular stresses and how these complex mitochondrial behaviors are coordinated at the molecular level remains elusive. It is important to address these questions, as mitochondrial dynamics and homeostasis are tightly linked with cellular physiology and eventually cell fate.

John Lemasters first termed selective mitochondrial autophagy as “mitophagy” (Lemasters, 2005). Mitophagy is a process that selectively sequesters damaged or depolarized mitochondria into double-membraned autophagosomes for subsequent lysosomal degradation. The removal of damaged or unwanted mitochondria, mitophagy was found to be essential for maintaining cellular fitness. Both ubiquitin- and receptor-mediated mitophagy pathways have been described and extensively studied. Intriguingly, BCL2 family proteins were reported to participate in both mitochondrial dynamics and mitophagic processes, which puts them in the center of mitochondrial homeostasis. We recently have shown that PGAM5, a mitochondrial phosphatase, serves as a molecular switch for determining mitochondrial fate (apoptosis or mitophagy) by dephosphorylating BCL-xL, a key apoptosis inhibitor and FUNDC1, a mitophagy receptor. These results demonstrated the integration of stress signals and the coordinated execution of graded responses in response to mitochondrial stress conditions (Ma K. et al., 2019). Here, we provide a focused overview on the molecular mechanisms of mitophagy and its interplay with mitochondrial dynamics and behaviors, thus contributing to aging and aging-related diseases.

## Molecular Regulation of Mitophagy

### Mitophagy in Yeast

Electron microscopy has revealed that, in *Saccharomyces cerevisiae*, mitochondria can be specifically sequestered by autophagosomes, or be engulfed together with cytosolic material (Kissova et al., 2007). This process depends on the complete set of Atg-proteins such as Atg11, Atg17, and Atg29, as well as specific adaptor proteins (Farre et al., 2009). The mitochondrial outer membrane protein, Uth1, and mitochondrial protein phosphatase homolog, Aup1, have both been implicated in mitophagy (Kissova et al., 2007; Tal et al., 2007). Pioneering work from Ohsumi’s and Klionsky’s laboratories have identified that, Atg32, a mitochondria-anchored protein, is essential for mitophagy in yeast. It acts as a mitophagy-specific receptor and interacts with autophagy key proteins such as Atg8 via an Atg8 interacting motif (AIM) and Atg11 to recruit autophagosomes to mitochondria for their engulfment and final degradation (Kanki et al., 2009; Okamoto et al., 2009). Atg32 undergoes both transcriptional and post-translational regulation in response to mitophagy induction. Expression of *Pichia pastoris* Atg32 (PpAtg32, Atg32 homolog in *P. pastoris*) is highly suppressed in nutrient-rich media caused by the DNA-binding protein Ume6 and the histone deacetylase complex Sin3–Rpd3, which interact with the promoter region of the gene encoding PpAtg32 to repress its transcription (Aihara et al., 2014). Kang’s group provided evidence that the kinase CK2 could phosphorylate N-terminal cytosolic region of Atg32 at serine 114 and serine 119 to promote the Atg32–Atg11 interaction and further accelerate the mitophagic process (Kanki et al., 2013), but how CK2-dependent phosphorylation takes place during starvation remains elusive. The C-terminal intermembrane space domain of Atg32 was found to be proteolytically processed by inner membrane i-AAA (ATPases associated with various cellular activities) protease Yme1 during mitophagy induction (Wang et al., 2013).

### Mitophagy in Mammalian System

It has become clear that the regulation of mitophagy in mammalian cells appears to be more complex. Thus, both ubiquitin-mediated and receptor-mediated pathways have been described to facilitate mitophagy in response to cellular, developmental, and environmental cues in mammalian systems.

#### Ubiquitin Pathways

In mammalian cells, the PTEN-induced putative kinase protein 1 (PINK1) and Parkin-mediated ubiquitination pathway is one of the most-studied mitophagy mechanisms so far. Two key factors, the serine/threonine kinase PINK1 and the E3 ubiquitin ligase Parkin, cooperatively sense cellular stress and mediate the removal of damaged mitochondria. Under physiological conditions with normal mitochondrial membrane potential, PINK1 is continuously imported into mitochondria where it is cleaved by the intramembrane protease presenilin associated rhomboid like (PARL), leading to its retro-translocation into the cytosol and rapid proteasomal degradation (Sekine and Youle, 2018). When mitochondrial membrane potential drops, PINK1 escapes from PARL-dependent cleavage and aggregates on the outer mitochondrial membrane to exert its pro-mitophagic function. Stabilized PINK1 phosphorylates both Parkin and ubiquitin (at Ser65) to promote ubiquitination of outer mitochondrial membrane proteins (Kane et al., 2014; Koyano et al., 2014). Phosphorylated ubiquitin binding to Parkin further unleashes Parkin from its autoinhibited state (Kazlauskaite et al., 2015). Activated Parkin appends ubiquitin moieties on specific mitochondrial outer membrane proteins such as MFN1, MFN2, FIS1, and translocase of outer mitochondrial membrane (TOMM) proteins, thus inducing their proteasomal degradation, which in turn promotes mitochondrial fission and mitophagy (Tanaka et al., 2010; Desai et al., 2018). The phosphatase and tensin homolog (PTEN)-long (PTEN-L) is able to dephosphorylate (Ser65 of) both ubiquitin and Parkin, which reduces the mitochondrial translocation of Parkin and negatively regulates mitophagy (Wang et al., 2018). On the other hand, the Parkin-mediated formation of ubiquitin chains on mitochondrial outer membrane proteins or even PINK1 itself can recruit ubiquitin-binding adaptor proteins such as optineurin (OPTN) and Calcium Binding And Coiled-Coil Domain 2 (CALCOCO2, better known as NDP52) onto mitochondrial surfaces, followed by the assembly of autophagy factors on Parkin and ubiquitin-marked mitochondria (Wong and Holzbaur, 2014; Lazarou et al., 2015). Ubiquitination of sperm mitochondria in both *Caenorhabditis elegans* and mammalian systems serves as “eat me” signal for their elimination by receptor-mediated mitochondrial degradation (Sutovsky et al., 1999; Molina et al., 2019). Both mitochondrial E3 ubiquitin protein ligase 1 (MUL1) and Parkin are necessary to remove paternal mitochondria from mouse embryos via mitophagy to ensure maternal mitochondrial inheritance (Rojansky et al., 2016).

Moreover, deubiquitinases play a crucial role in modulating the efficiency of PINK1 and Parkin-mediated mitophagy. Thus, ubiquitin-specific peptidase 8 (USP8) directly deubiquitinates Parkin and removes non-canonical Lys6-linked ubiquitin chains from Parkin, thereby promoting its translocation to depolarized mitochondria. In contrast to USP8 (Durcan et al., 2014), USP15 deubiquitinates the mitochondrial substrates of Parkin to inhibit mitophagy (Cornelissen et al., 2014). Recently, several deubiquitinases such as USP30, USP35, and USP33 were reported to antagonize Parkin-mediated ubiquitination and thus oppose Parkin-mediated mitophagy (Bingol et al., 2014; Wang et al., 2015; Niu et al., 2019). In addition, PINK1 and Parkin have been suggested to be required for mitochondria-derived vesicle (MDV)-dependent mitophagy such that vesicles budding from mitochondria under oxidative stress can be delivered to the lysosomes independent of LC3 (Soubannier et al., 2012; McLelland et al., 2014).

#### Mitophagy Receptor Pathway

Several mitophagy receptors have been identified in mammalian cells, significantly advancing the field of both mitochondrial and selective autophagy. Mitophagy receptors in mammalian cells are characterized by the presence of at least one LC3 interacting region (LIR) that can directly bind to the autophagy mediator LC3 to recruit autophagosomes to mitochondria.

BCL2 interacting protein 3 like (BNIP3L, better known as NIX) was identified as an essential mitophagy receptor for the autophagic clearance of mitochondria during the maturation of erythroid cells (Sandoval et al., 2008). Recently, the phosphorylation of the LIR domain of NIX was shown to further enhance the affinity of the interaction between NIX and LC3 (Rogov et al., 2017). Moreover, BCL2 interacting protein 3 (BNIP3), a homolog of NIX, was found to mediate mitophagy in conditions of hypoxia (Quinsay et al., 2010).

We have discovered that FUNDC1 acts as an important mitophagy receptor, whose function is regulated by its phosphorylation state (Liu et al., 2012). Structural analysis revealed the functional importance of the close proximity of Tyr18 of FUNDC1 with Asp19 of LC3. Consistently, phosphorylation of Tyr18 of FUNDC1 via SRC proto-oncogene, non-receptor tyrosine kinase (SRC) kinase significantly weakens its binding affinity for LC3 due to electrostatic repulsion *in vitro* (Kuang et al., 2016). The dephosphorylation (of Ser13) of FUNDC1 can promote mitophagy by recruiting Drp1 while dissociating it from OPA1, thus inducing mitochondrial fission (Chen et al., 2016).

Other mitophagy receptors have been reported such as BCL2 Like 13 (BCL2L13) (the functional homolog of ATG32 in mammals) (Otsu et al., 2015), FKBP prolyl isomerase 8 (FKBP8) (Bhujabal et al., 2017), NLR family member X1 (NLRX1) (Zhang Y. et al., 2019), autophagy and Beclin 1 regulator 1 AMBRA1 (Strappazzon et al., 2015), as well as the mitochondria inner membrane protein prohibitin 2 (PHB2) (Wei et al., 2017). All of them were found to interact with LC3 via the conserved LIR motif to mediate mitophagy when mitochondria become damaged. However, the molecular regulation and their cooperation in response to mitochondrial stresses are not completely understood. Moreover, mitophagy receptors are not limited to proteins, as certain types of lipids such as cardiolipin and ceramide have been reported to interact with LC3 and to mediate mitophagy (Sentelle et al., 2012; Chu et al., 2013).

### The Interplay Between Mitochondrial Dynamics and Mitophagy

Distinct molecular machineries have been identified to regulate mitochondrial fission and fusion. In mammalian cells, the GTPase MFN1, MFN2, and OPA1 mediate the fusion of the outer and inner membranes of mitochondria, respectively. Mitochondrial fission is regulated by Drp1 that normally resides in the cytosol and is recruited to mitochondria by mitochondrial fission factors such as FIS1, MFF, MIEF1, or MIEF2 (Mishra and Chan, 2014). ER tubules, which are in contact with mitochondria, play an active role in the initial step of mitochondrial division and mediate mitochondrial constriction before Drp1 recruitment (Friedman et al., 2011). At the final step of mitochondrial division, the Drp1-mediated constriction promotes dynamin-2 (DNM2) assembly, which can induce membrane fission to complete division (Lee et al., 2016). In response to bioenergetic crisis and oxidative stress, these mediators of mitochondrial dynamics are posttranslationally modified to fine-tune their activities. Phosphorylation of Drp1 by protein kinase A (PKA, also known as cAMP-dependent protein kinase) at Ser637 (Chang and Blackstone, 2007) and Ser656 (Cribbs and Strack, 2007) inhibits Drp1, resulting in mitochondrial elongation, while dephosphorylation of Drp1 at Ser65 by the calcium-dependent protein phosphatase calcineurin or by protein phosphatase 2A (PP2A) enhances mitochondrial fragmentation (Cribbs and Strack, 2007). Another report suggested that Drp1 is phosphorylated at Ser616 by the cyclin-dependent kinase 1 (CDK1)/cyclin B complex during mitosis (Taguchi et al., 2007; Marsboom et al., 2012). The phosphorylation of the Drp1 receptor MFF by energy-sensing adenosine monophosphate (AMP)-activated protein kinase (AMPK) results in the recruitment of Drp1 and final mitochondrial fragmentation (Toyama et al., 2016). Other Drp1 modifications include *S*-nitrosylation (Cho et al., 2009) and ubiquitination by MARCH5 to mediate mitochondrial division (Karbowski et al., 2007) or by Parkin to inhibit mitochondrial fission (Wang et al., 2011).

The mitochondrial fusion molecule MFN1 can be phosphorylated by extracellular regulated kinase (ERK) at Thr562 to inhibit fusion (Pyakurel et al., 2015), ubiquitinated by MARCH5 (Park et al., 2014), and deubiquitinated by USP30 (Yue et al., 2014) to regulate protein stability and fusion activity, while MFN2 can be phosphorylated by mitogen-activated protein kinase 8 (MAPK8 better known as JNK) at Ser27 and ubiquitinated for degradation by HUWE1 (Leboucher et al., 2012), Parkin (Gegg et al., 2010), and MARCH5 (Sugiura et al., 2013), and deubiquitinated by USP30 (Yue et al., 2014). During mitophagy, MFN2 also functions as a mitochondrial receptor for the PINK1-dependent recruitment of Parkin. PINK1 phosphorylates MFN2 at Thr111 and Ser442 to promote the recruitment of Parkin to depolarized mitochondria (Chen and Dorn, 2013). OPA1 can be proteolytically processed by mitochondria-resident proteases, including YME1-like 1 ATPase (YME1L) (Griparic et al., 2007) and zinc metallopeptidase (OMA1) (Head et al., 2009), in response to intra-mitochondrial signals, to regulate fusion of the inner mitochondrial membrane.

It was suggested that mitochondrial fission is necessary for mitochondrial degradation by mitophagy because fission enables the separation of depolarized mitochondria from the mitochondrial network and allows their engulfment by autophagosomes. Mitochondrial stress-induced mitophagy is accompanied by enhanced mitochondrial fission. The inhibition of mitochondrial fission processes by overexpression of dominant negative Drp1^K38A^ or knockdown of FIS1 decreases mitophagy and leads to the accumulation of oxidized mitochondrial proteins (Twig et al., 2008). In agreement with this, mitophagic players were found to regulate mitochondrial dynamics. Thus, Parkin is able to ubiquitinate MFN1 and MFN2 to promote their degradation, leading to increased fragmentation of mitochondria (Gegg et al., 2010). Our early work showed that Parkin also ubiquitinates and degrades Drp1 (Wang et al., 2011). This may be counterintuitive, as degradation of Drp1 prevents mitochondrial fragmentation. It is possible that under homeostatic conditions, Parkin monitors the molecular status of Drp1 to prevent mitochondrial fragmentation, and upon stress conditions, Parkin translocates to mitochondria to promote mitochondrial fragmentation and mitophagy.

Mitophagy receptors such as FUNDC1 and BNIP3 were found to promote mitochondrial fission in response to stress (Landes et al., 2010; Chen et al., 2016). FUNDC1 directly interacts and recruits Drp1 toward mitochondria for mitochondrial fission. Interestingly, FUNDC1 is a transmembrane protein with a motif that faces the mitochondrial intermembrane space and directly interacts with OPA1 to promote mitochondrial fission.

It was noted that mitochondrial fission is necessary, but not sufficient for mitophagy. Reports suggested that Drp1-mediated mitochondrial fission was dispensable for mitophagy (Song et al., 2015; Yamashita et al., 2016). We have found that mitochondrial targeting of the LIR-containing cytosolic portion of FUNDC1 is sufficient to induce mitophagy even in the absence of mitochondrial fragmentation, when phosphorylation of Tyr18 is blocked (Kuang et al., 2016). Recently, by using structure illumination microscopy (SR-SIM), Xian et al. (2019) observed that the overexpression of the SNARE protein syntaxin 17 (STX17) initiated mitophagy in FIS1-depleted cells but not in other mitochondria dynamic factors-silenced cells. They further demonstrated that FIS1 negatively regulated STX17 by inhibiting its trafficking to mitochondria-associated membranes (MAMs) and mitochondria independent of mitochondrial dynamics (Xian et al., 2019). In summary, a sensitive reaction to various types of stress mitochondrial fragmentation at early stages facilitates segregation and clearance of dysfunctional mitochondria from the mitochondrial network for maintaining mitochondrial and cellular homeostasis.

### Mitochondrial Dynamics and Cell Death

Mitochondria in mammalian cells sense apoptotic stress, mainly through BCL2 and its family proteins, ultimately leading to MOMP and the subsequent release of cytochrome c and other apoptogenic factors for the activation of the caspase cascade governing apoptotic cellular disintegration. The BCL2 protein family is composed of antiapoptotic molecules including BCL2, BCL-xL, MCL1, and proapoptotic molecules such as BCL2 associated X (BAX), BCL2 antagonist/killer 1 (BAK), and BH-3-only subfamily proteins such as such as BIM, BAD, NOXA, and BID (Doerflinger et al., 2015). In healthy cells, BAX and BAK1 are blocked by antiapoptotic proteins such as BCL2, BCL-xL, and MCL1, which contain four BH motifs (BH1–4). The BH3-only proteins can induce apoptosis by direct interaction with BAX and BAK or by binding to antiapoptotic members and thus neutralizing the inhibitory sequestration of BAX and BAK (Chen et al., 2005; Chipuk et al., 2010). The antiapoptotic protein BCL-xL interacts with BAX to continuously retro-translocate mitochondrial BAX into the cytosol and keep it from integrating into the mitochondrial outer membrane (Edlich et al., 2011). In apoptotic cells, BAX and BAK oligomerization triggers MOMP and initiates the caspase cascade ultimately leading to cell death (Tait and Green, 2010).

Emerging evidence indicates that the mechanisms governing mitochondrial dynamics are also involved in the regulation of apoptotic processes. Inhibition of mitochondrial fission reduces cytochrome c release and apoptosis (Frank et al., 2001; Cereghetti et al., 2010). On the contrary, the dephosphorylation of Drp1 at Ser637 by the phosphatase calcineurin promotes Drp1-mediated mitochondrial fragmentation and leads to apoptosis (Cereghetti et al., 2010). Drp1-dependent mitochondrial fission through MIEF2 facilitates apoptotic cristae remodeling during the early phase of intrinsic apoptosis (Otera et al., 2016), and moreover, Drp1 can stimulate truncated Bid (tBID)-induced Bax oligomerization and cytochrome c release by promoting tethering and hemifusion of membranes. Dephosphorylation of Drp1 by the mitochondrial phosphatase PGAM5 can facilitate necroptosis by enhancing mitochondrial fission (Wang et al., 2012). However, other reports have shown that blocking mitochondrial fission can just delay but does not block apoptosis (Parone et al., 2006; Rolland and Conradt, 2010; Clerc et al., 2014).

On the other hand, BCL2 family members can affect the morphology of mitochondria. In healthy cells, BAX and BAK are required for mitochondrial fusion (Karbowski et al., 2006). Mitochondria are fragmented and have less network continuity in cells lacking BAX and BAK. The interaction between BAX and MFN2 activates the assembly of the MFN2 complex and changes its membrane mobility and distribution. However, the activation of pro-apoptotic BAX and BAK promotes the fragmentation of the mitochondrial network during apoptosis (Autret and Martin, 2009; Montessuit et al., 2010), which is not inhibited by the expression of BCL-xL, MCL1, or other members of the BCL2 subfamily (Sheridan et al., 2008). BAX and BAK form foci that colocalize with ectopic MFN2 and Drp1 at the sites of mitochondrial division to promote mitochondrial fission during apoptosis (Karbowski et al., 2002). Furthermore, BCL-w, an antiapoptotic BCL2 family member, was proposed to regulate mitochondrial fission in Purkinje cell dendrites (Liu and Shio, 2008). BCL-xL overexpression induces the remodeling of the mitochondrial network by altering the relative rates of mitochondrial fusion and fission (Delivani et al., 2006; Li et al., 2008). In neuronal cells, overexpression Bcl-xL can increase the rates of both fission and fusion and mitochondrial biomass (Berman et al., 2009). Other studies found that, in hippocampal neurons, BCL-xL increases synapse dynamics and the localization of mitochondria to synapses and vesicle clusters via a Drp1-dependent manner (Li et al., 2013). Alternatively BCL-xL can interact with Drp1 to function in mitochondrial fission during neuronal development (Li et al., 2008). Although the mechanisms of action require further clarification, these findings demonstrate that the BCL2 protein family indeed orchestrates mitochondrial morphology and apoptosis.

### Mitophagy and Cell Death

The BCL2 family was initially recognized for their function in apoptosis, and is now widely proven to also have other roles in cellular function involving mitochondrial dynamics, autophagy/mitophagy, and cellular metabolism. Early studies have shown that the antiapoptotic protein BCL2 can interact with Beclin 1 (BECN1) to inhibit autophagy (Pattingre et al., 2005). Further analysis reveals that depending on its phosphorylation status, BCL2 has dual roles in regulating autophagy and apoptosis. It suggests that initial JNK1-mediated BCL2 phosphorylation may promote cellular survival by disrupting BCL2–BECN1 complexes and activating autophagy (Wei et al., 2008a). At a point when autophagy is no longer able to maintain survival, the phosphorylation of BCL2 serves to inactivate its antiapoptotic function for progression of regulated cell death (Wei et al., 2008b). Parkin-dependent mitophagy is antagonized by BCL-xL and MCL1 in a BECN1-independent manner. Specifically, BCL2 and BCL-xL suppress Parkin translocation to depolarized mitochondria, while BH3-only proteins (or BH3-only mimetics) can promote this process (Hollville et al., 2014).

Several mitophagy receptors including BNIP3, NIX, and BCL2L13 belong to the BCL2 family (Novak et al., 2010; Hanna et al., 2012; Murakawa et al., 2015), highlighting an intrinsic link of mitophagy with apoptosis. Apparently, these BCL2 family proteins have dual roles in both apoptosis and mitophagy. For example, BNIP3 and NIX can directly interact with antiapoptotic BCL2 or BCL-xL, which antagonizes the activation of proapoptotic BAX and BAK, to promote apoptosis (Imazu et al., 1999; Dorn, 2010). As discussed above, NIX also induces mitophagy via its interaction with LC-3, and enhanced interaction of BNIP3 with Atg8 family members promotes pro-survival mitophagy prior to cytochrome c release and apoptosis (Zhu et al., 2013). It was also found that the mitochondrial fragmentation is a prerequisite for BNIP3-induced mitophagy in cardiac myocytes, and dominant negative Drp1^K38E^ mutant, or MFN1 overexpression inhibit BNIP3-induced mitochondrial division and mitophagy (Lee et al., 2011). Similar to NIX, BNIP3 induces the disintegration of elongated mitochondria into numerous spherical particles, accompanied by the recruitment of Drp1 to fragmented mitochondria in adult myocytes (Lee et al., 2011). Moreover, BNIP3 can directly interact with OPA1, promote the disassembly of OPA1 oligomers, and thus antagonize its fusion activity in HeLa cells (Landes et al., 2010). Thus, BCL2 family proteins act as general regulators of mitochondrial dynamics and homeostasis, in addition to their role in apoptosis-associated mitochondrial permeabilization.

Mitophagy was suggested to play a protective role in stress-induced cell death and early studies showed that Parkin strongly inhibits the translocation of BAX to mitochondria, thus preventing apoptosis (Darios et al., 2003; Johnson et al., 2012). Further studies revealed that Parkin is able to directly ubiquitinate the apoptotic effector proteins such as BAX and BAK, and the ubiquitination of BAK by Parkin impairs its activation and the formation of oligomers to suppress errant apoptosis (Bernardini et al., 2019). Parkin suppression of BAX-dependent apoptosis will allow the effective clearance of apoptotic mitochondria to limit their potential pro-inflammatory effect (Bernardini et al., 2019). Parkin suppression of apoptosis is likely the cellular context and apoptosis inducer dependent. Studies from Seamus Martin’s laboratory showed that upon mitochondrial depolarization, the BCL2 family member MCL1 underwent rapid PINK1- and Parkin-dependent polyubiquitination and degradation, which sensitized cells toward apoptosis via opening of the BAX and BAK-dependent pathway. Knockdown of BAX is able to suppress Parkin-dependent apoptosis in HeLa cells (Carroll et al., 2014). It was also reported that NIX-mediated mitophagy protects glioblastoma cells against hypoxia (Jung et al., 2019). Furthermore, abrogating NIX- and FUNDC1-mediated mitophagy during adult cardiac progenitor cells (CPCs), differentiation leads to increased susceptibility to cell death (Lampert et al., 2019).

In addition, there is certain evidence showing that mitophagy plays an accelerative role in programmed cell death. Thus, LC3–ceramide interactions provoked by ceramide treatment induce mitophagy and can progress to autophagic cell death in human cancer cells (Sentelle et al., 2012). Moreover, inhibition of mitophagy and mitochondrial fission reduces cigarette smoke-induced necroptosis in mice epithelial cells *in vitro*, and in chronic obstructive pulmonary disease (COPD) *in vivo* (Mizumura et al., 2014). Furthermore, in hippocampal neural stem cells deprived of insulin, Parkin-mediated mitophagy is necessary for autophagy-dependent cell death (Park et al., 2019). Moreover, drug-induced mitochondrial dysfunction and heme oxygenase 1 (HMOX1) overactivation synergize to trigger lethal mitophagy in glioma cells, which is significantly blocked by silencing of the mitophagy receptors BNIP3 and NIX (Meyer et al., 2018).

**FIGURE 1:**
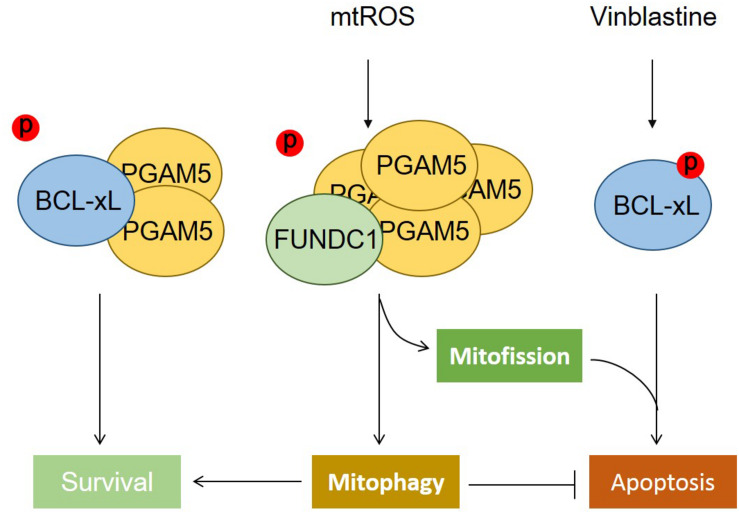
The phosphatase PGAM5 regulates mitochondrial fate. The phosphatase PGAM5 is a dimeric protein that can bind with and dephosphorylate BCL-xL at Ser62, which increases its antiapoptotic function and thus inhibits apoptotic cell death. Mitochondrial oxidative stress causes the transformation of dimeric PGAM5 into a multimeric state that fails to bind with BCL-xL, but instead interacts with and dephosphorylates FUNDC1 at Ser13 to mediate mitochondrial fission and mitophagy. The dephosphorylation of FUNDC1 cooperates with the phosphorylation of BCL-xL, aggravating cell death when mitophagy is blocked.

We have found that BCL-xL, but not BCL2, strongly suppresses FUNDC1-mediated mitophagy. BCL-xL interacts with and inhibits the mitochondrial Ser/Thr phosphatase PGAM5 to prevent the dephosphorylation of FUNDC1 (at Ser13), thus further blocking hypoxia-induced mitophagy (Wu et al., 2014). The functions of PGAM5 not only are limited to the induction of mitophagy but also involve the regulation of mitochondrial homeostasis (Figure 1). PGAM5 exists in an equilibrium between a dimeric and a multimeric state, which is sensitive to oxidative stress. Dimeric PGAM5 binds with and dephosphorylates BCL-xL in mitotically arrested cells, thus exerting its antiapoptotic function *in vitro* and *in vivo*. Mitochondrial oxidative stress enhances the multimerization of PGAM5, resulting in its dissociation from BCL-xL. Liberated multimeric PGAM5 dephosphorylates FUNDC1 to initiate mitochondrial fission and mitophagy. When FUNDC1-mediated mitophagy is blocked by the microtubule inhibitor vinblastine, PGAM5 dephosphorylates FUNDC1 and mediates mitochondrial fission that aggravates vinblastine-induced cell death (Ma K. et al., 2019).

### Mitophagy Is Balanced With Mitochondrial Biogenesis for Mitochondria Homeostasis

Mitophagy is balanced with mitochondrial biogenesis, together defining mitochondrial turnover. Mitochondrial biogenesis is a cellular process in which “new” mitochondria are produced, depending on the cooperation of nuclear and mitochondrial genome (Zhang and Xu, 2016). Mitochondrial biogenesis preserves mitochondrial function and cellular homeostasis (Rasbach and Schnellmann, 2007; Miwa et al., 2008; Gottlieb and Carreira, 2010), while mitophagy mitigates the source of oxidative stress that reduces the risk of apoptosis (Hickson-Bick et al., 2008). The crosstalk between mitophagy and mitochondrial biogenesis also allows cells to undergo metabolic reprogramming during development and differentiation. Similar to mitophagy, mitochondrial biogenesis is highly variable and tightly regulated in response to diverse stimuli such as energy demand, cell cycle, and intracellular stress (Zhang and Xu, 2016). Many cellular signaling pathways converge on the regulation of both mitophagy and mitochondrial biogenesis such as the mammalian target of rapamycin (mTOR), which regulates cellular growth and energy homeostasis in conditions of nutrient stress (Dibble and Cantley, 2015; Vyas et al., 2016). The mTOR signal pathway can transcriptionally and translationally regulate mitochondrial biogenesis. Thus, mTOR controls mitochondrial function through the modulation of PPARG coactivator 1 alpha (PGC1α) transcriptional activity (Cunningham et al., 2007) and ablation of PPARGC1B (PGC1β) is associated with the constitutive activation of mTORC1 (Camacho et al., 2012). At the transcriptional level, it can mediate the activation of PGC1α, which is a key transcriptional co-activator regulating mitochondrial biogenesis via its interaction with a variety of transcription factors (Morita et al., 2015). Similar to mTOR, the hypoxia-inducible factor 1 subunit alpha (HIF1A) signaling in response to limited oxygen availability can impinge on mitochondrial biogenesis, mitophagy, and mitochondrial metabolism through regulation of PGC1α (LaGory et al., 2015).

mTORC1 phosphorylates UNC-51 like autophagy activating kinase 1 (ULK1) and 2 (ULK2) and disrupts the interaction between ULK1 and protein kinase AMP (AMPK), leading to the inhibition of autophagy and mitophagy when nutrient levels are sufficient. Under nutrient starvation, ATP depletion leads to serine/threonine kinase 11 (LKB1)-mediated AMPK activation (Garcia and Shaw, 2017), which in turn leads to the phosphorylation of ULK1 to initiate autophagy and mitophagy (Kim et al., 2011; Tian et al., 2015). Tuberous sclerosis complex (TSC1/2) is an inhibitor of the mTOR signaling pathway. In TSC1/2-deficient neurons, axonal and global mitophagy is impaired and mitochondrial homeostasis can be restored by blocking mTORC1 (Ebrahimi-Fakhari et al., 2016). Moreover, TSC2-deficient cells exhibit constitutive mTOR activation, impaired autophagic flux, and accumulation of damaged mitochondria, which associates with reduced PINK1 expression and Parkin mitochondrial translocation to mitochondria. These data link mTOR signaling to PINK1-Parkin-mediated mitophagy (Bartolome et al., 2017).

Hypoxia-induced mtROS can activate HIFs leading to the upregulation of target genes, including the mitophagy receptor BNIP3 (Bell et al., 2007; Chourasia and Macleod, 2015). As a feedback mechanism, BNIP3-mediated mitophagy reduces the generation of mtROS, which in turn can stabilize HIF1A (Chourasia and Macleod, 2015). Nevertheless, ROS can also activate JNK–PGC1α signaling pathway to promote mitochondrial biogenesis and the expression of genes involved in OXPHOS (Chae et al., 2013). Increased mtROS was also found to promote cellular proliferation by activating NF-κB (Guha et al., 2010). Recently, we found that a hypoxic microenvironment can induce siah E3 ubiquitin protein ligase 2 (SIAH2)-dependent ubiquitination and subsequent proteasomal degradation of nuclear respiratory factor 1 (NRF1), a transcription factor crucial for mitochondrial biogenesis. In conditions of cancer, this signaling axis alters the level of mitochondrial biogenesis and is involved in metabolic adaptations finally maintaining tumor progression (Ma B. et al., 2019).

## Mitochondrial Quality Control in Immune Responses

The innate and adaptive immune systems are able to sense pathogen-associated molecular patterns (PAMPs) and danger-associated molecular patterns (DAMPs) arising from exogenous clues including bacteria, virus, fungi, and parasites, as well as endogenous entities such as cancer cells and to mount defensive immune responses. Mitochondria have emerged as central organelles contributing to immune response at multiple levels, such as adaptations of mitochondrial metabolism, dynamics, biogenesis, and mitophagic turnover. Mitochondrial DNA (mtDNA), once released into the cytoplasm, acts as intrinsic DAMP, which can be sensed by toll-like receptor 9 (TLR9) and triggers nuclear factor kappa B (NF-κB) signaling in human polymorphonuclear neutrophils (Zhang et al., 2010). Moreover, mtDNA activates the NLRP3 inflammasome, which in turn boosts the production of cytokines such as IL18 and IL1β induces pyroptosis in immune cells (Liu et al., 2018). Furthermore, cellular mtDNA activates the STING pathway via cGAS and leads to the expression of IRF3-dependent genes such as type I interferons and participates to antiviral immune responses (West et al., 2015). Interestingly, live-cell lattice light-sheet microscopy observed mouse embryonic fibroblasts result has shown that BAK/BAX form macropores after activation and allowed mitochondrial matrix components, including the mtDNA releasing into the cytosol (McArthur et al., 2018). In addition, evidence has shown that mtROS enhances the NLRP3 inflammasome activation and upregulates NF-κB signaling. Mitophagy counteracts chronic inflammation via the elimination of damaged mitochondria, which are the major sources of mtDNA and ROS.

Parkin-mediated mitophagy restrains excess ROS and cytosolic mtDNA, and inhibits NLRP3 inflammasome activity in macrophages and favors tissue repair via a NF-κB and sequestosome 1 (SQSTM1, better known as p62)-dependent mitophagic pathway (Zhong et al., 2016). Intriguingly, Parkin is cleaved by caspase-1 to limit mitophagy and resultant excess inflammation (Yu et al., 2014). Defective mitophagy leads to the upregulation mRNA levels of inflammasome-related proteins in primary hepatocytes under palmitic acid treatment and in a murine model of NASH (Zhang N. P. et al., 2019). Defects in mitophagy arising from deletion, mutation, or silencing of mitophagy receptors, such as NIX, Parkin, and p62, lead to mitochondrial dysfunctions and have been linked to inflammasome activation and cancer (Drake et al., 2017). Likewise, ablation of FUNDC1 causes the inhibition of mitophagy and increases the accumulation of dysfunctional mitochondria, which in turn results in inflammasome activation and inflammatory responses that can promote hepatocyte tumorigenesis *in vivo* (Li et al., 2019). *Listeria monocytogenes* can induce mitophagy in macrophages to evade host immune response. Mechanistically, Nod-like receptor (NLR) family member X1 (NLRX1), a novel mitophagy receptor located at the mitochondria, directly interacts with LC3 via its LIR motif, thus contributing to the induction of mitophagy for the elimination of ROS, and maintains the survival of *L. monocytogenes* (Zhang Y. et al., 2019). Furthermore, interleukin 10 (IL-10), an anti-inflammatory cytokine that promotes mitophagy, leads to a decrease in the activation of the NLRP3 inflammasome and the production of IL-1β in macrophages (Ip et al., 2017).

The mitochondrial outer membrane is a platform for MAVS-mediated innate immune responses, which are activated by the viral RNA sensors RIG-I-mediated signaling cascade culminating in the activation by NF-κB and IRF3 (Seth et al., 2005). Alternatively, MAVS can oligomerize upon sensing mtROS independent of RIG-I to facilitate the production of type I interferon (Buskiewicz et al., 2016). Intriguingly, MAVS has been found to contain a LIR motif and act as a potential receptor for mitophagy (Sun et al., 2016). Furthermore, the ubiquitination of MAVS by ring finger protein 34 (RNF34) causes NDP52-associated mitophagy to mitigate innate immune response upon viral infection (He et al., 2019).

BCL2 family regulated mitochondria-dependent cell death has also been reported to play an important role in innate and adaptive immune responses. One of the therapeutic strategies to Legionnaires’ disease is the pharmacological inhibition of BCL-xL. Inhibition of BCL-xL can induce the apoptosis of macrophages infected with virulent Legionella and thus abrogate Legionella replication and disease progression in mice (Speir et al., 2016). Additionally, it is well known that activated T cells will undergo cell death once the antigen has disappeared. This mechanism is triggered by the BCL-xL- and BCL2-mediated release of pro-apoptotic BAX and BAK or the fact that BCL-xL without its unstructured loop, which cannot bind to any form of BAX and BAK, binds BIM less well than wild-type BCL-xL and thus sensitizes T cells to the induction of regulated cell death (Liu et al., 2006). Overall, more and more evidence demonstrates that mitochondrial quality control mechanisms regulate essential functions in immune cell and are important in controlling immune responses. Mitophagy plays a protective role in cellular homeostasis to negatively regulate innate immune response, but a systematic drawing of this intricate relationship has not been completed yet due to its complexity.

## Dysregulation of Mitochondrial Homeostasis in Aging and Aging-Related Diseases

Aging increases the risk for the onset of various chronic diseases often associated with the accumulation of mtDNA mutations, altered mitochondrial mass, compromised mitochondrial functions, chronic immune activation, and accelerated cell death. These pathogenic manifestations are likely due to dysregulated mitochondrial dynamics and mitochondrial quality control mechanisms, leading to the accumulation of dysfunctional mitochondria, which enhances both chronic immune activation (through the release of mtDNA and ROS) and mitochondrial apoptosis (through the liberation of apoptogenic factors). There is emerging evidence that compromised mitophagy causes aging, while enhancing mitophagy by caloric restriction and physical exercises increases healthy life span. Thus, the reduction of mitophagy has been suggested as areas on for the accumulation of mitochondria in aged *C. elegans* (Palikaras et al., 2015), and the induction of mitophagy results in life span extension in this model (Ryu et al., 2016). Overexpression of the *Drosophila* PGC-1 homolog (dPGC-1/spargel) increases mitochondrial activity, and intestinal stem cell (ISC) lineage-specific expression of dPGC-1 leads to an extended life span in *Drosophila melanogaster* (Rera et al., 2011). Physical exercise caused the activation of AMPK that leads to ULK1 phosphorylation and enhanced mitophagy in skeletal muscle, which in turn promotes mitochondrial turnover and improves general health in murine models (Laker et al., 2017).

Mitochondrial dysfunction is a common pathogenic factor for neurodegenerative disorders. Mitochondria supply ATP, generate mtROS, and regulate calcium homeostasis, all of which affect neuronal cell physiology. Aberrations in mitochondrial ROS and Ca^2+^ homeostasis have been implicated in Parkinson’s disease (PD) (Ludtmann and Abramov, 2018). Both elevated cytosolic Ca^2+^ levels and mitochondrial ROS are pathological hallmarks of PD. Thus, PINK-1 deficiency in midbrain neurons leads to mitochondrial Ca^2+^ overload in response to dopamine, which further promotes ROS production and neuronal cell death (Gandhi et al., 2009). The loss of mitochondrial fission factor (MFF) increases mitochondrial size and mitochondrial Ca^2+^ uptake during neurotransmission, thus affecting neurotransmitter release and neuronal fitness (Lewis et al., 2018). Production of mtROS caused by damaged mitochondria in mice microglia promotes the secretion of pro-inflammatory cytokines and results in neurodegeneration (von Bernhardi et al., 2015). Furthermore, the accumulation of Ca^2+^ and ROS in the mitochondria triggers mitochondrial permeability transition pore (mPTP) opening, subsequently releasing cytochrome c and other pro-apoptotic intermembrane space proteins into the cytosol (Hunter and Haworth, 1979). It needs to be noted that BCL2 family proteins are involved in the regulation of Ca^2+^ dynamics of the ER and the mitochondria (Vervliet et al., 2016). For instance, a fraction of NIX is localized at the conjunction between mitochondria and the ER to regulate ER and mitochondrial Ca^2+^ homeostasis (Diwan et al., 2009). In addition, the upregulation of NIX in cardiac hypertrophy was associated with the apoptotic death of cardiomyocytes (Yussman et al., 2002). Altogether, mitochondrial quality control appears crucial for protecting neurons from damage and death.

It has been well established that compromised mitophagy contributes to the pathogenesis of Parkinson’s disease, and enlarged or swollen mitochondria have been observed in several disease models and in the brains of Parkinsonian patients. Mutations in PINK1 and Parkin are involved in rare familial cases of Parkinson’s disease (PD) (Kitada et al., 1998; Valente et al., 2004). PGAM5 deficiency disables PINK1-mediated mitophagy *in vitro* and causes a Parkinson’s-like phenotypes in mice model (Lu et al., 2014; Sekine et al., 2016). It is still puzzling that *Parkin* knockout mouse does not completely recapitulate PD phenotype. When Parkin knockout mouse was crossed with mouse that harbors high mtDNA mutation, the accumulation of mutated mtDNA in neuronal cells was observed, but these mice does not have much increase of mitochondrial mass (Gautier et al., 2008; Stevens et al., 2015; Pinto et al., 2018). Studies using the recently developed mitophagy reporter mice and Drosophila also show that mitophagy is rather constitutive and is minimally impacted by loss of PINK1 or Parkin (McWilliams et al., 2016; Whitworth and Pallanck, 2017; Lee et al., 2018; McWilliams et al., 2018), suggesting that additional factors are required in the absence of PINK1 or Parkin. Mitophagy has been implicated with disease progression in Alzheimer’s disease (Witte et al., 2009; Wilhelmus et al., 2011). Thus, Alzheimer’s disease phenotype-related accumulation of mutant amyloid beta precursor protein (APP) induces Parkin-dependent mitophagy in cultured human neurons and in the brain of Alzheimer’s patients (Ye et al., 2015). Recent studies showed that Tau pathology, another hallmark of Alzheimer’s disease, impairs mitophagy by inhibiting Parkin translocation to mitochondria (Cummins et al., 2019). These studies indicate that insufficient mitophagy might be the cause for the accumulation of damaged mitochondria in Alzheimer’s disease-affected neurons. Conversely, there are arguments that mitophagy improves the neuropathology of Alzheimer’s disease and reverses cognitive deficits in animal models (Kerr et al., 2017).

Moreover, recent studies have suggested that the loss of mitophagy regulators is closely linked to cardiovascular disease. Thus, Parkin-mediated mitophagy is required for the metabolic transition in the perinatal murine heart (Gong et al., 2015). Deletion of PINK1, Parkin, or other mitophagy receptors such as FUNDC1, BNIP3, or NIX leads to the accumulation of dysfunctional mitochondria and results in various heart defects involved in exacerbated ischemia/reperfusion injury and cardiomyopathy (Dorn, 2010; Kubli et al., 2013; Zhang et al., 2016). Consistently, impaired PINK1 and Parkin-mediated mitophagy affected by Parkin deficiency or mutations in MFN2 results in the retention of fetal cardiac mitochondria, reduced oxidative metabolism, heart failure, and premature death (Chen and Dorn, 2013; Gong et al., 2015), highlighting that mitophagy underlies mitochondrial plasticity and metabolic transitioning in developing cardiomyocytes.

## Summary and Future Perspectives

**FIGURE 2:**
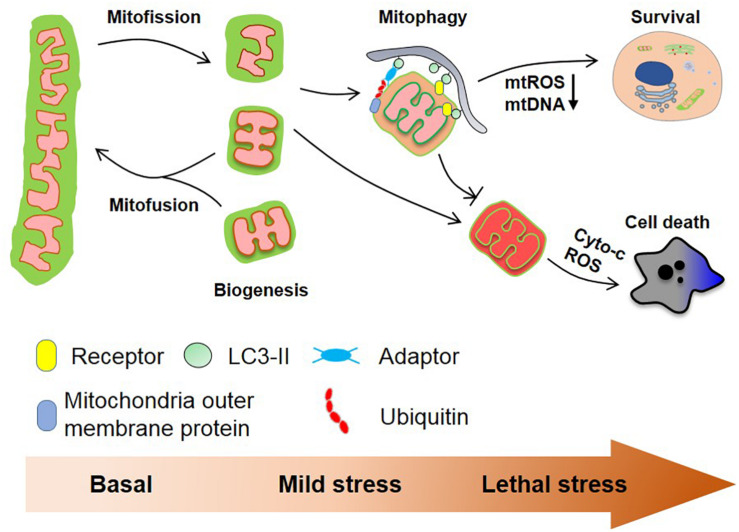
Mitochondrial homeostasis and cell fate. Mitochondria are dynamic organelles that constantly divide and fuse in healthy cells and mitochondrial biogenesis and mitophagy cooperate to maintain mitochondrial quality. Mitochondrial fission usually is a prerequisite for ubiquitin- and receptor-mediated mitophagy facilitating the removal of aged and damaged mitochondria. Severe stress-induced mitochondrial damage can lead to mitochondria outer membrane permeabilization (MOMP) that in turn triggers apoptosis. The clearance of depolarized mitochondria by mitophagy (before MOMP occurs) mitigates this process. Besides, mitophagy reduces the production of mtROS and the cytosolic secretion of mtDNA, which further prevents excess immune response.

Mitochondria are highly plastic organelles that adapt to cellular and environmental stress and developmental cues by changes in their morphology and their overall mass. Changes in the mitochondrial behaviors are regulated by distinct but interlinked molecular machineries that control mitochondrial dynamics (fission, fusion) and mitochondrial homeostasis (mainly through biogenesis and mitophagy), collectively allowing the graded response to stress (Figure 2). BCL2 family proteins, and in particular BCL-xL, act as global regulators of mitochondrial homeostasis and quality control through their interaction with various partners including DRP1, MFN1/2, and PGAM5, that are tightly controlled by reversible phosphorylation, acetylation, and ubiquitination, thereby modulating mitochondrial behaviors and cell fate. This is further exemplified by our recent finding that the mitochondrial phosphatase, PGAM5 exists in an equilibrium between a dimeric and a multimeric state to dephosphorylate FUNDC1 and BCL-xL, respectively, to switch on/off mitophagy and apoptosis. Further research is needed to explore the (patho-) physiological roles of this molecular switch in response to environmental and cellular stresses.

By removing the damaged and unwanted mitochondria, mitophagy is essential for mitochondrial quality control and homeostasis. As discussed above, almost all mitophagy players including both receptor-dependent pathway and PINK1/Parkin pathway are found to regulate mitochondrial dynamics and apoptosis. Furthermore, mitophagy not only governs the mitochondrial quality and quantity, but also controls mitochondrial dynamics and behaviors. Enhanced mitophagy and mitochondrial turnover contributes to increased mitochondrial function and cellular activity. Conversely, the inhibition of mitophagy leads to accelerated aging and the manifestation of aging-associated diseases. Moreover, the accumulation of dysfunctional mitochondria, and the associated release of mtDNA, the overproduction of mtROS, and mitochondria-controlled apoptosis result in a chronic state of immune activation, which is also the common etiology for aging-associated neurodegenerative disease. We further suggest that targeting mitophagy is an important strategy to fight aging and aging-associated disease, which needs to be further explored in the future.

## Author Contributions

KM, GC, QC, and WL wrote the manuscript. OK and YZ revised the manuscript. All authors provided intellectual input and read the manuscript.

## Conflict of Interest

OK is one of the co-founders of Samsara Therapeutics, Inc. The remaining authors declare that the research was conducted in the absence of any commercial or financial relationships that could be construed as a potential conflict of interest.
